# Identification and analysis of the complete mitochondrial genome of *Acheilognathus omeiensis* (Cypriniformes, Cyprinidae)

**DOI:** 10.1080/23802359.2018.1443032

**Published:** 2018-02-26

**Authors:** Yuanchao Zou, Mei Chen, Tian Wu, Tian Tian, Zhengyong Wen

**Affiliations:** a College of Life sciences, Conservation and Utilization of Fishes resources in the Upper Reaches of the Yangtze River Key Laboratory of Sichuan Province, Neijiang Normal University, Neijiang, Sichuan, China;; b School of Life Sciences, Southwest University, Chongqing, China

**Keywords:** *Acheilognathus omeiensis*, Mitochondrial genome, phylogenetic analysis

## Abstract

*Acheilognathus omeiensis* is a small-size freshwater ornamental fish. In this study, the complete mitochondrial genome sequence of *A. omeiensis* was first determined. This mitogenome was 16,774 bp in length and contained 13 protein-coding genes (PCGs), 22 tRNA genes, 2 rRNA genes, and 2 main noncoding regions. Most mitochondrial genes were encoded on the H-strand, except for ND6 and eight tRNA genes. The base composition was 28.97% A, 27.72% T, 26.02% C, and 17.29% G, with 56.69% AT, respectively. Phylogenetic tree was constructed based on the nucleotide sequence of 13 PCGs of *A. omeiensis* and closely related 12 species ticks to assess their phylogenic relationship and evolution. These data should be helpful for a better understanding of the mitochondrial genomic diversities and evolution in fish as well as novel genetic markers for studying population genetics and species identification.

*Acheilognathus omeiensis* (Cyprinidae, Acheilognathinae) is a small-size freshwater ornamental fish. It is distributed in the upper stream of Yangtze River and its tributary (Zhu et al. 2006). There was less report about its basic biology data including genetic information. In this study, the complete mitochondrial DNA sequence was first determined by the next generation sequencing (NGS).

The specimens were obtained from Qingjiang town of Jintang County, Sichuan Province of China (30°55′6.08″N, 104°22′49.21″E), in September 2017 and were stored in Zoological Specimen Museum of Neijiang Normal University (accession number: 20170915BB02). A 30–40 mg fin clip was collected and preserved in 95% ethanol at 4 °C. Total genomic DNA was extracted with a Tissue DNA Kit (OMEGA E.Z.N.A.) following the manufacturer’s protocol. Subsequently, the genomic DNA was sequenced using the NGS, and then the mitogenome was assembled using *A. macropterus*as reference.

The complete mitochondrial genome of *A. omeiensis* was a circular molecule with 16,774 bp long (GenBank Accession number MG783572). It consisted of 13 protein-coding genes (PCGs), 22 tRNA genes, 2 rRNA genes, a D-loop control region, and an origin of replication on the light-strand (OL). The overall base composition of *A. omeiensis* was 28.97% A, 27.72% T, 26.02% C, and 17.29% G, with a total AT content of 56.69%, which was consistent with other teleost mitogenomes (Hwang et al. 2012; Zou et al. 2017a). The 12 PCGs started with a common initiation codon ATG and the remaining COI with GTG, which was similar with *A. somjinensis* and *A. macropterus* (Hwang et al. 2014; Zhu et al. 2014). Moreover, most PCGs terminated with TAA codon except four genes (ND2, COII, COIII, Cyt b) with incomplete codon T-, one gene (ND4) with TA- and two genes (ND3, ND6) with TAG codon, which was different from *A. barbatus* (ND4 with T–, ND5 with TAG, ND6 with TAA) (Tao and Sun 2014). Most of the mitochondrial genes of *A. omeiensis* encoded on H-strand except for ND6 and eight tRNA genes (tRNAGln, tRNAAla, tRNAAsn, tRNACys, tRNATyr, tRNASer, tRNAGlu, tRNAPro). The 22 tRNA genes were interspersed among the whole genome ranging in size from 68 (tRNACys) to 76 bp (tRNALeu, tRNALys). The 12S rRNA (958bp) and 16S rRNA(1,677bp) were located between tRNAPhe and tRNALeu and separated by the tRNAVal gene. There were several gene overlaps, such as the open reading frames of ATP8-ATP6, ATP6-COIII, ND4L-ND4, and ND5-ND6. In addition, the largest interval (OL, 31 bp) was located between tRNAAsn and tRNACys.

Thus far, the mitochondrial PCGs have been widely used for inferring phylogenetic relationships (Boore et al. 2005). Based on the nucleotide alignments of 13 PCGs, the phylogenetic tree inferred using different methods (ML and NJ) was identical in both datasets with high Bootstrap values (Zou et al. 2017b). *Channa argus* was used as outgroup. Both trees showed that *Acheilognathus* was divided into two monophyletic clades (A, B) and *A. imberbis*, *A. intermedia*, *A. signifer,* and *A. somjinensis* were grouped into clade B, and the rest of the species were clustered into clade A (Figure 1). Furthermore, two topologys also showed that *A. omeiensis* and sister group (*A. macropterus*, *A. chankaensis, A. barbatus* and *A. typus*) had a close relationship, and further confirmed that *A. omeiensis* belonged to Acheilognathinae subfamily, which was in line with the morphological classifcation.

**Figure 1.**
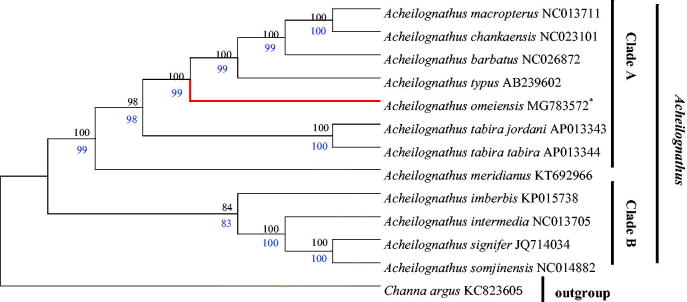
Phylogenetic relationship between 13 taxa. The phylogenetic tree was constructed using maximum likelihood (ML) and neighbor-Joining (NJ) method based on the nucleotide alignments of 13 protein-coding genes. The tree topologies produced by ML and NJ analyses were equivalent. NJ posterior probabilities (black number) and ML bootstrap values (blue number) are shown on the nodes. *Channa argus* (GenBank: KC 823605) was used as the outgroup.
